# Therapeutics

**Published:** 1879-09

**Authors:** 


### THERAPEUTICS.

CHOLAGOGUES.—(*Br. Med. Jour.*, Feb. 8., '79.)

For some time a commission, with Dr. Rutherford, of Edinburg at its head, has been investigating the biliary secretion of the dog, with reference to the action of cholagogues. Having at last found itself able to give a summary of results, we give a few of its conclusions concerning the actions of well known remedies, calling attention, however to the fact that they were obtained on dogs, and may not be exactly similar with the human race.

Podophyllin, aloes, rheum, euonymin, sanguinarin, iridin, leptandrin, colocynth, jalap, sodium phosphate, mercuric chloride, phytolaccin, hydrastin, juglandin, sodium and ammonium benzoate, benzoic acid, sodium salicylate and nitro-muriatic acid, all are more or less powerful hepatic stimulants, positively increasing the amount of bile secreted, and all except the last five being more or less stimulant or irritating to the intestinal glands. Senna, colchicum, taraxacum and scammony are but feeble hepatic stimulants. Gamboge, castor oil and calomel stimulate the intestinal glands, but not the liver. Ipecacuanha is simply a hepatic stimulant. Magnesium sulphate is only stimulant to the intestines, as are also ammonium chloride and menispermin.

Calabar bean stimulates the liver, and atropia antagonizes this action, but atropia given alone does not affect the secretion of bile.

The authors have also found, in man, four grains of iridin, at night, a certain remedy for "biliousness." Euonymin, in two grain doses, also has the same effect. Both are liable to be followed by depression, if the dose be too large, and both should be followed by a saline aperient in the morning.

